# Comparative profiling of osimertinib and fourth-generation EGFR inhibitors on EGFR-mutant proteins and cell lines

**DOI:** 10.3389/fonc.2026.1952701

**Published:** 2026-09-21

**Authors:** Janneke J. T. M. Melis, Kirsten J. W. Kevenaar, Jacob Ytsma, Daphne J. F. Kluitmans, Janneke M. van den End, Jeffrey J. Kooijman, Jonne L. K. van Eck, Esmee van den Bossche, Jeroen A. D. M. de Roos, Yusuke Kawase, Nicole Willemsen-Seegers, Yvonne Grobben, Guido J. R. Zaman

**Affiliations:** 1 Oncolines B.V., Oss, Netherlands; 2 Carna Biosciences, Inc., Kobe, Japan

**Keywords:** drug resistance, EGFR, kinase inhibitor, non-small cell lung cancer, osimertinib, surface plasmon resonance

## Abstract

**Background:**

Osimertinib is a third-generation, irreversible EGFR inhibitor that targets common activating EGFR mutations (ex19del and L858R) and the resistance EGFR mutation T790M. Clinical resistance to osimertinib frequently arises through the EGFR C797S mutation, which has driven the development of fourth-generation EGFR inhibitors.

**Methods:**

We performed side-by-side biochemical and cellular profiling of osimertinib and three fourth-generation EGFR inhibitors: BLU-945, JBJ-09-063 and silevertinib. Kinase assays were performed both at *K*_m,ATP_ and at physiological ATP concentration (1 mmol/L). Effects on cell viability were evaluated in a CRISPR-engineered NCI-H1975 cell line harboring L858R + T790M + C797S and across a 113 cancer cell line panel including ten *EGFR*-mutant cell lines, harboring mutations other than C797S.

**Results:**

Biochemical assays revealed distinct profiles among the fourth-generation EGFR inhibitors across different EGFR variants under *K*_m,ATP_ and 1 mmol/L ATP concentrations. BLU-945 was the only inhibitor demonstrating broad activity across activating EGFR variants with or without the T790M and/or C797S resistance mutation. JBJ-09-063 selectively inhibited L858R-driven mutants and maintained similar potency across ATP conditions, which is consistent with an allosteric, ATP-independent mechanism. Silevertinib showed high potency against activating EGFR mutants with or without the C797S mutation but markedly reduced activity in the presence of the T790M mutation. All fourth-generation inhibitors retained binding to C797S mutants in surface plasmon resonance experiments. Moreover, JBJ-09-063 bound EGFR L858R + T790M even after osimertinib saturation, confirming their non-overlapping binding sites. In the engineered NCI-H1975 L858R + T790M + C797S cell line model, JBJ-09-063 showed the highest potency among the tested inhibitors. In contrast, silevertinib demonstrated the greatest mutant-selective activity across a broad cell-line panel, with >200-fold selectivity for EGFR-mutant over EGFR wild-type cells, outperforming osimertinib in this regard.

**Conclusion:**

This comparative analysis highlights the substantial differences in the activity landscapes of fourth-generation EGFR inhibitors. The gathered insights can provide direction for the optimal deployment of the inhibitors in distinct clinical contexts.

## 1 Introduction

Approximately one-third of patients with non-small cell lung cancer (NSCLC) harbor an activating mutation in the epidermal growth factor receptor (*EGFR*) gene. The two most frequently observed activating *EGFR* mutations are a leucine-to-arginine substitution at position 858 (L858R) and in-frame deletions in exon 19 (ex19del) (1). The activating mutations lead to constitutive receptor signaling and activation of downstream pathways, resulting in uncontrolled cell growth and tumorigenesis (2).

In 2004, it was discovered that NSCLC patients harboring either activating *EGFR* mutation had significantly greater benefit from gefitinib than patients without such mutation (3, 4). These findings established selective targeting of *EGFR*-mutant NSCLC with small-molecule kinase inhibitors as a central therapeutic paradigm for this deadly disease. However, as with nearly all cancer therapies, the emergence of drug resistance is inevitable and has driven the development of successive generations of EGFR inhibitors. Each new generation has led to improvements in progression-free and overall survival for patients with *EGFR*-mutated NSCLC (5–7). These advances have contributed to a substantial decline in NSCLC mortality, from 26.4 per 100,000 individuals in 2000 to 17.1 per 100,000 in 2020, according to the Global Cancer Observatory (https://gco.iarc.fr/en, accessed on 26 June 2026). Despite this progress, NSCLC remains one of the deadliest cancers worldwide, according to the World Health Organization (www.who.int, accessed 26 June 2026).

Osimertinib (**Figure 1**) is a third-generation EGFR inhibitor that irreversibly binds to a cysteine residue (C797) in the active site of the EGFR kinase domain (8). It effectively targets EGFR with activating mutations and the resistance-associated T790M mutation that emerges following treatment with first- or second-generation EGFR inhibitors (8) (**Table 1**). Osimertinib was initially approved as second-line treatment in 2015, but following on positive results in a phase 3 clinical trial, the FDA approved osimertinib in 2018 for first-line treatment of metastatic NSCLC with common activating EGFR mutations (5, 9–12). However, despite the durable clinical responses observed, resistance to osimertinib inevitably emerges over time (13), highlighting the need for the development of fourth-generation EGFR inhibitors. The most common on-target resistance mutation to osimertinib is a cysteine-to-serine substitution at position 797 (C797S), which prevents the covalent binding of osimertinib to the ATP-binding pocket of EGFR (13).

**Figure 1:**
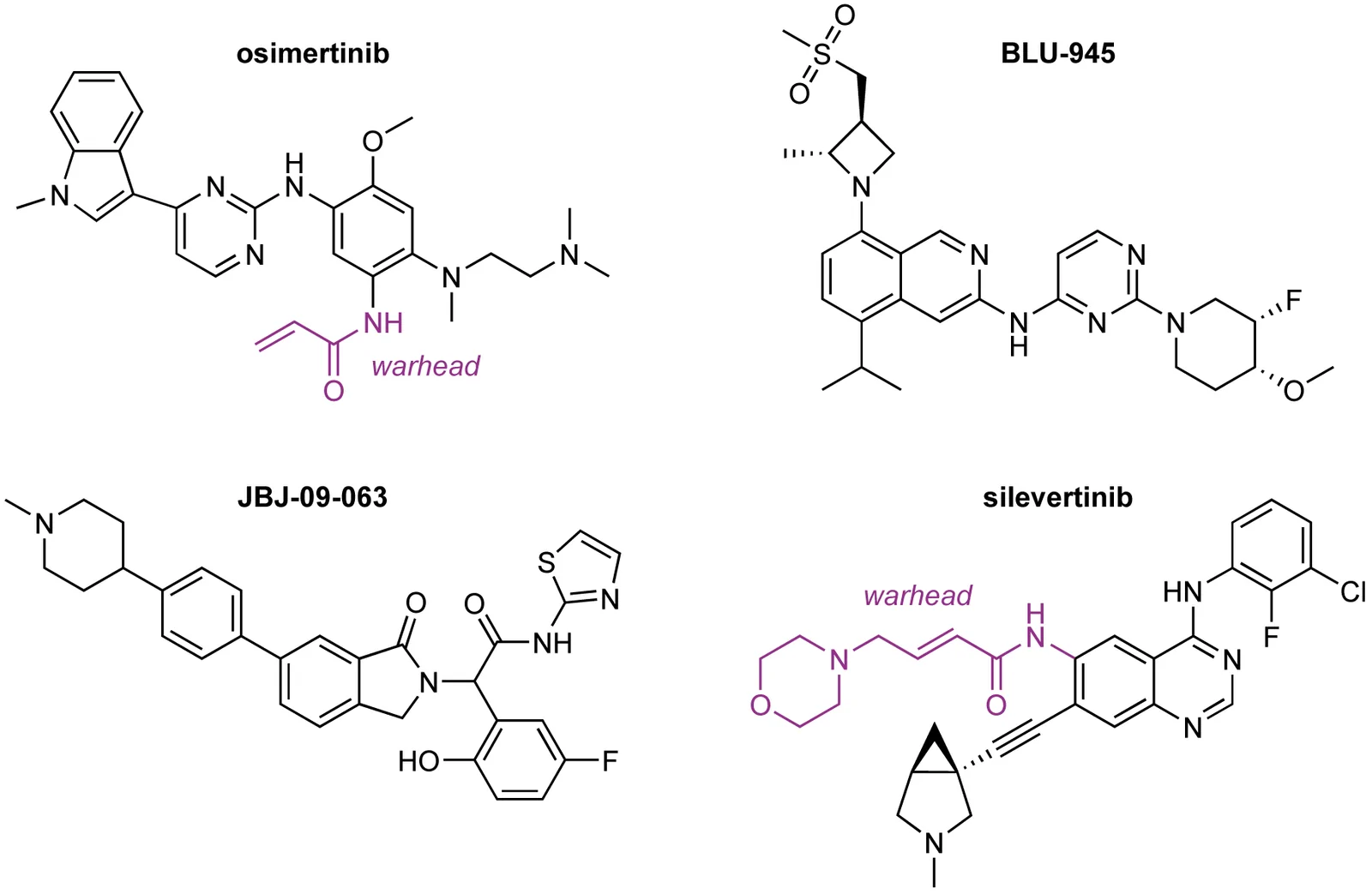
Chemical structures of osimertinib and the three fourth-generation EGFR inhibitors studied: BLU-945, JBJ-09-063 and silevertinib. The reactive chemical group (‘warhead’) within the structures of the covalent inhibitors osimertinib and silevertinib are indicated in purple.

**Table 1:** Overview of profiled EGFR inhibitors.

Inhibitor	Generation	Binding mode	Binding site	Target indication (EGFR)	Development status	Reference
osimertinib	Third	Irreversible	Orthosteric	L858R/ex19del + T790M	Marketed	Cross et al. (8)
BLU-945	Fourth	Reversible	Orthosteric	L858R/ex19del + T790M + C797S	Discontinued	Eno et al. (14)
JBJ-09-063	Fourth	Reversible	Allosteric	L858R + T790M + C797S	Investigational	Beyett et al. (15)
silevertinib	Fourth	Irreversible	Orthosteric	Various classical and non-canonical mutations	Phase 1/2	Flohr et al. (17)

Currently, several fourth-generation EGFR inhibitors are in development. In this study, we investigated three fourth-generation EGFR inhibitors, that is, BLU-945, JBJ-09-063 and silevertinib (BDTX-1535) (**Figure 1**, **Table 1**). BLU-945 is an orthosteric, reversible inhibitor developed to target either activating EGFR mutations L858R or ex19del in combination with T790M or T790M + C797S, while sparing wild-type EGFR (14). JBJ-09-063 is an allosteric inhibitor that stabilizes the inactive conformation of EGFR (15, 16). Its binding site is shaped by the EGFR L858R driver mutation and its potency is not affected by resistance mutations located in the ATP-binding site (15, 16). Silevertinib is designed to target both common and uncommon EGFR mutants, including those harboring the C797S mutation (17). Although silevertinib is currently in clinical development, available data on its potency and efficacy have predominantly been presented in conference abstracts and press releases, with limited peer-reviewed literature available to date (18–23).

The here described fourth-generation EGFR inhibitors are in different phases of development. However, public data remain limited, and differences in experimental models and methodologies make it difficult to compare the inhibitors both to osimertinib and to one another. To enable extensive comparison of the EGFR inhibitors, we investigated osimertinib and the three fourth-generation inhibitors side-by-side in biochemical and cell-based assays.

## 2 Methods

### 2.1 Inhibitors

Osimertinib and silevertinib were purchased from MedKoo BioSciences, Inc. (Durham, NC, USA). BLU-945 and JBJ-09-063 were purchased from MedChemExpress LLC (Monmounth Junction, NJ, USA). The purity and identity of all compounds were verified using liquid chromatography–mass spectrometry. The purity of all compounds was > 98%. The inhibitors were stored as dry powders at 4 °C and were dissolved in dimethyl sulfoxide (DMSO) at a final concentration of 10 mmol/L.

### 2.2 Kinase assays

The cytoplasmic domain of EGFR and its mutant variants were expressed in Sf21 insect cells and purified at Carna Biosciences, Inc. (Kobe, Japan) (24, 25). All constructs extended from amino acid position 669 to the C-terminus (position 1210). The proteins are listed in **Supplementary Table 1**. Kinase inhibition was determined using mobility shift assays (MSA) with a duplicate 10-point inhibitor dilution series at Carna as previously described (24). Assays were conducted with a reaction time of one hour and at two ATP concentrations: (i) at *K*_m,bin_, that is, at an ATP concentration within two-fold of the enzyme’s Michaelis constant for ATP (24) (this condition is further referred to in the manuscript as *K*_m,ATP_), and (ii) at a concentration of 1 mmol/L ATP. The apparent Michaelis constants of the different variants, and the concentrations of enzyme and ATP used in the kinase assays are given in **Supplementary Table 1**. For the EGFR L858R + T790M + C797S mutant, no kinase assay is available at *K*_m,ATP_, since this assay did not meet predefined assay criteria.

### 2.3 Surface plasmon resonance

Binding kinetics were studied on a Biacore T200 and 1S+ (Cytiva, Uppsala, Sweden) (26). Biotinylated EGFR L858R (cat. no. 08-402-20N), EGFR L858R + C797S (cat. no. 08-763-20N) and EGFR L858R + T790M (cat. no. 08-410-20N) were produced at Carna Biosciences, Inc. (Kobe, Japan). The proteins were immobilized on streptavidin-coated sensor chips (Cytiva, cat. no. BR100531) to an immobilization level of about 4000 resonance units (RU), and unbound streptavidin was subsequently blocked with biocytin. Immobilizations were performed at 4 °C with 50 mmol/L Tris (pH 7.5), 0.05% (v/v) Tween-20, 150 mmol/L NaCl and 5 mmol/L MgCl_2_ as the running buffer, whereas subsequent assays were performed at 22 °C with running buffer supplemented with 1% (v/v) DMSO. A 30-minute pre-run at a flow rate of 30 µL/min was performed to obtain a stable surface. Binding kinetics of EGFR inhibitors were measured using single-cycle kinetics with five sequential compound injections of increasing concentrations (0.1–10, 1–100, 10–1,000 or 100–10,000 nmol/L), depending on the inhibitor affinity. The association phase lasted 100 seconds per concentration, followed by a dissociation phase of 1200 seconds. A flow rate of 30 µL/min was maintained to minimize mass transport limitations. Binding data were double-referenced using blank runs preceding each compound dilution series and using an empty reference channel without protein.

Resulting sensorgrams were fitted to appropriate binding models using Biacore T200 or Insight Evaluation Software (Cytiva) to determine kinetic binding parameters, which were geometrically averaged across experiments. A 1:1 Langmuir binding model was used for non-covalent inhibitors (Equation 1).

(1) $$P+I\;\begin{array}{c} \begin{array}{c} k_{a} \end{array}\\ ⇌\\ \begin{array}{c} k_{d} \end{array} \end{array}\;PI$$

Here, *P* represents the protein, while *I* represents the inhibitor. The equilibrium dissociation constant (*K*_D_), representing the binding affinity of the inhibitor, was calculated from the association and dissociation rate constant (*k*_a_ and *k*_d_) using the equation *K*_D_ = *k*_d_/*k*_a_. For covalent inhibitors (*i.e.*, osimertinib and silevertinib on EGFR variants without the C797S mutation), a modified two-state reaction model was used. The regular two-state reaction model provided by the software describes the 1:1 binding of inhibitor to immobilized protein followed by a reversible stabilizing change (Equation 2).

(2) $$P+I\;\begin{array}{c} \begin{array}{c} k_{a1} \end{array}\\ ⇌\\ \begin{array}{c} k_{d1} \end{array} \end{array}\;PI\;\begin{array}{c} k_{a_{2}}\\ ⇌\\ k_{d2}\; \end{array}\;P\cdot I$$

PI represents the non-covalent protein–inhibitor complex, whereas P·I represents the complex that has undergone a reversible stabilizing change. In the modified model, the reverse rate constant of the stabilizing change (*k*_d2_) was set to zero to reflect the irreversibility of the covalent bond formation and the maximal rate of inactivation (*k*_inact_) was determined as the forward rate constant of the stabilizing change (*k*_a2_) (27) (Equation 3).

(3) $$P+I\;\begin{array}{c} \begin{array}{c} k_{a} \end{array}\\ ⇌\\ \begin{array}{c} k_{d} \end{array} \end{array}\;PI\begin{array}{c} k_{inact}\\ \to \end{array}P*I$$

P*I represents the irreversible, covalent complex formed upon formation of a covalent bond between the protein and inhibitor. The inactivation constant (*K*_I_), representing the covalent inhibitor concentration yielding a half-maximal rate of inactivation (½*k*_inact_), was calculated using the equation *K*_I_ = (*k*_d_ + *k*_inact_)/*k*_a_. Each protein-inhibitor combination was measured in at least two independent experiments, or in a single experiment with at least three technical replicates.

### 2.4 Cell lines

Human cancer cell lines were obtained from the American Type Culture Collection (ATCC, Manassas, VA, USA), German Collection of Microorganisms and Cell Cultures (DSMZ, Braunschweig, Germany), RIKEN BioResource Research Center (Tsukuba, Ibaraki, Japan), and Japanese Collection of Research Bioresources (JCRB, Ibaraki City, Osaka, Japan). All cell lines were cultured in the media as indicated in **Supplementary Table 2**. Cell viability assays were conducted within ten passages of the original vials. The authenticity of the ATCC and DSMZ cell lines was verified by short tandem repeat (STR) analysis performed by the respective institutions. Additionally, the mutation status of several cancer genes has been confirmed in multiple cell lines through next-generation sequencing of genomic DNA derived from the same cell batches as used in the viability assays (28, 29).

### 2.5 Generation of a C797S-mutant lung cancer cell line

The EGFR C797S mutation was introduced into the NSCLC cell line NCI-H1975 (EGFR L858R + T790M) using CRISPR/Cas9 gene editing at Synthego (Redwood City, CA, USA) (**Supplementary Table 3**). Cells from the transfected pool were seeded as single cells using an SH800 fluorescence-activated cell sorter (Sony, Weybridge, United Kingdom) and were expanded into three clones. The presence of the C797S mutation in the clones was confirmed by whole-exome sequencing (WES) using Illumina NovaSeq 6000 sequencing. Data analysis was performed using the DRAGEN platform (version 3.10). The frequencies of the EGFR L858R, T790M and C797S mutations in the parental cell line and the three cell clones are summarized in **Supplementary Table 4**.

### 2.6 Immunoblotting

The abundance of total EGFR and phosphorylated EGFR in gene-edited NCI-H1975 cell clones and parental cells was analyzed by SDS-PAGE, followed by immunoblotting. Total EGFR was detected using an anti-EGFR (D38B1) XP^®^ rabbit monoclonal antibody (Cell Signaling, cat. no. 4267S, RRID: AB_2246311), followed by an HRP-conjugated anti-rabbit IgG secondary antibody (Cell Signaling, cat. no. 7074S. RRID: AB_2099233). The membranes were stripped using Restore™ Plus Western Blot Stripping Buffer (Thermo Fisher, cat. no. 46430) and subsequently re-probed with a phospho-specific antibody targeting EGFR phosphorylated at Tyr1068 (Cell Signaling, cat. no. 2234S, RRID: AB_331701), followed by the HRP-conjugated anti-rabbit IgG to detect phosphorylated EGFR. To confirm equal loading across samples, β-actin was detected using a rabbit anti-β-actin polyclonal antibody (Cell Signaling, cat. no. 4967, RRID: AB_330288). Antibody binding was detected by enhanced chemiluminescence (ECL) using Clarity Western ECL substrate (BioRad, cat. no. 1705060) and visualized using a BioRad ChemiDoc imaging system. Blots were quantified using ImageJ. Full blots are shown in **Supplementary Figure 1**.

### 2.7 Flow cytometry

Cell-surface EGFR expression in gene-edited NCI-H1975 clones and parental cells was quantitatively measured using a Quantibrite Phycoerythrin (PE) Fluorescence Quantitation Kit (BD Biosciences, cat. no. 340495, RRID: AB_2868736) on a Guava^®^ easyCyte 12 HT flow cytometer (Cytek Biosciences B.V., Amsterdam, The Netherlands). Cultured cells were washed, detached using trypsin-EDTA (Thermo Fisher, cat. no. 25300054) and counted. One million cells were resuspended in autoMACS^®^ Running Buffer (Miltenyi Biotec, cat. no. 130-091-221), containing 0.5% bovine serum albumin, 2 mmol/L EDTA and 0.09% sodium azide. Cells were stained for 30 minutes at 4 °C in the dark with 5 µg/mL PE-labeled anti-EGFR antibody clone AY13 (BioLegend, #352904, RRID: AB_10896794) or 10 µg/mL PE-labeled mouse IgG1, κ isotype control antibody clone MOPC-21 (BioLegend, #400114, RRID: AB_326435). Following staining, the cells were washed with autoMACS^®^ Running Buffer and resuspended in SYTOX Red Dead Cell Stain (Thermo Fisher, cat. no. S34859) diluted 1:1000 in autoMACS^®^ Running Buffer. Flow cytometry data were collected from at least three independent experiments, with all samples measured in duplicate. Data were analyzed using Kaluza Analysis Software (Beckman Coulter). Gating strategies were employed to exclude debris, identify single viable cells, and select for EGFR-positive cells. For each sample 30,000 events were recorded. Quantibrite beads labeled with four defined levels of PE were used to generate a standard curve, which was used to calculate the average number of antibodies bound per cell via linear regression.

### 2.8 Quantitative real-time PCR

Total RNA was extracted from gene-edited and parental NCI-H1975 cell cultures using an RNeasy Mini Kit (Qiagen, cat. no. 74104). cDNA synthesis was performed using a QuantiTect Reverse Transcription Kit (Qiagen, cat. no. 205311). Quantitative real-time PCR (qPCR) was conducted on a Bio-Rad CFX96 cycler using TaqMan Master Mix (Thermo Fisher, cat. no. 444557). TaqMan probes were used to detect *EGFR* (Thermo Fisher, Assay ID Hs01076090_m1) and the reference gene *ACTB* (β-actin) (Thermo Fisher, Assay ID Hs01060665_g1) for normalization. The relative *EGFR* expression was calculated using the 2^-ΔΔCq^ method (30). qPCR was performed in three independent experiments and samples were measured in technical duplicates.

### 2.9 Cell viability assays

Intracellular ATP content was used as an indirect readout of cell number using the ATPlite 1Step bioluminescence assay (Revvity, Groningen, The Netherlands). The cells were seeded in 384-well plates at optimized densities to ensure unrestricted growth and maximal signal intensity at the end of the assay. After 24 h of incubation, the starting cell number was determined by adding ATPlite to each well of a control plate and luminescence was measured using an EnVision multimode plate reader (Revvity, Waltham, MA, USA) (31, 32). Inhibitors were serially diluted in DMSO using √10-fold steps from 10 mmol/L stock solutions to generate 9-point dilution series. These series were further diluted 31.6-fold in 20 mmol/L HEPES buffer (pH 7.4) and added in duplicate to the cells in 384-well plates. Vehicle-treated wells (0.4% DMSO, v/v) served as controls to determine the maximum cell growth. Following incubation for an additional 72 h, ATP content in each well was measured using ATPlite. Luminescence signals from the compound-treated wells were normalized to those from vehicle-treated controls to calculate the percentage of viable cells at each concentration. Cell doubling was determined by comparing the luminescence of vehicle-treated wells with the starting cell number for each cell line. Assays were repeated when the cell lines showed a greater than two-fold deviation from the historical doubling rate. For most cell lines, doubling rates were determined from more than hundred independent experiments. To monitor assay quality, a parallel test using doxorubicin was performed on one reference cell line. Half-maximal inhibitory concentration (IC_50_) values were calculated by fitting a four-parameter logistic model to the viability data using IDBS XLfit5 (IDBS, Guildford, United Kingdom). All dose-response curves were visually inspected and statistically assessed using an F-test (as implemented in XLfit5); curves with F-values exceeding 1.5 were considered unreliable. IC_50_ values were maximized at the highest tested concentration. The initial concentration range for each inhibitor spanned from 31.6 µmol/L to 3.16 nmol/L. When this range proved too high for reliable determination of the IC_50_ in specific cell lines, a new dilution series was generated from a diluted stock solution, and the assay was repeated accordingly.

### 2.10 Bioinformatics

The 113 cancer cell lines used in the cell viability assays were annotated as *EGFR* wild-type or *EGFR* mutant, resulting in a total of 10 *EGFR* mutant cell lines (**Supplementary Table 3**). For five of the *EGFR* mutant cell lines, the *EGFR* mutation and corresponding variant allele frequency were previously confirmed by in-house DNA sequencing (28) (**Supplementary Table 5**). The *EGFR* mutation status of the remaining *EGFR* mutant cell lines was annotated as in the DepMap database (OmicsSomaticMutations, release 24Q2) (**Supplementary Table 5**) (33, 34). To ensure that alterations included in the analyses are relevant to cancer growth and drug response, only mutations were included in the analyses that are either reported as a hotspot mutation in Cancer Hotspots; described as oncogenic, gain-of-function, or involved in drug response in OncoKB (35, 36); or described as pathogenic in the literature. Notably, we identified an A289V substitution in RL95-2, which has not been reported in the DepMap database. This is most likely due to its low variant allele frequency of 0.12 (28). The *EGFR* amplification status of the *EGFR*-mutant cell lines was annotated using the DepMap Copy Number WGS Public database (OmicsCNGeneWGS, release 25Q3) (34, 37). Z-scores were determined for the *EGFR*-mutant cell lines that were included in the database and scores > 2 were annotated as amplification. Cell viability assay data were expressed as ^10^logIC_50_ values in nmol/L. The ^10^logIC_50_ values of the inhibitors were compared between the *EGFR*-mutant and *EGFR* wild-type cell line groups. A Wilcoxon rank-sum test was performed to determine the significance of the differences in sensitivity between the two groups. A *p*-value < 0.05 was considered significant. The statistical analysis was performed in R (version 4.3.1) (38).

### 2.11 Statistical analysis

One-way analyses of variance (ANOVA) were performed to assess differences in RNA and surface protein expression levels between groups. Following a significant ANOVA result, Tukey’s *post-hoc* test was used for pairwise comparisons of the groups. A *p*-value < 0.05 was considered significant. Statistical analyses were performed using GraphPad Prism (version 11.0.0) (Dotmatics, Boston, MA, USA).

## 3 Results

### 3.1 Biochemical characterization in enzyme activity assays

The potency of osimertinib and the three fourth-generation EGFR inhibitors (**Table 1**) was determined in biochemical kinase activity assays with wild-type and mutant EGFR proteins at two ATP concentrations: *K*_m,ATP_ and 1 mmol/L ATP, with the latter reflecting intracellular concentrations (39–41). These assays revealed a distinct biochemical profile for each inhibitor (**Figure 2A**, **Table 2**).

**Figure 2:**
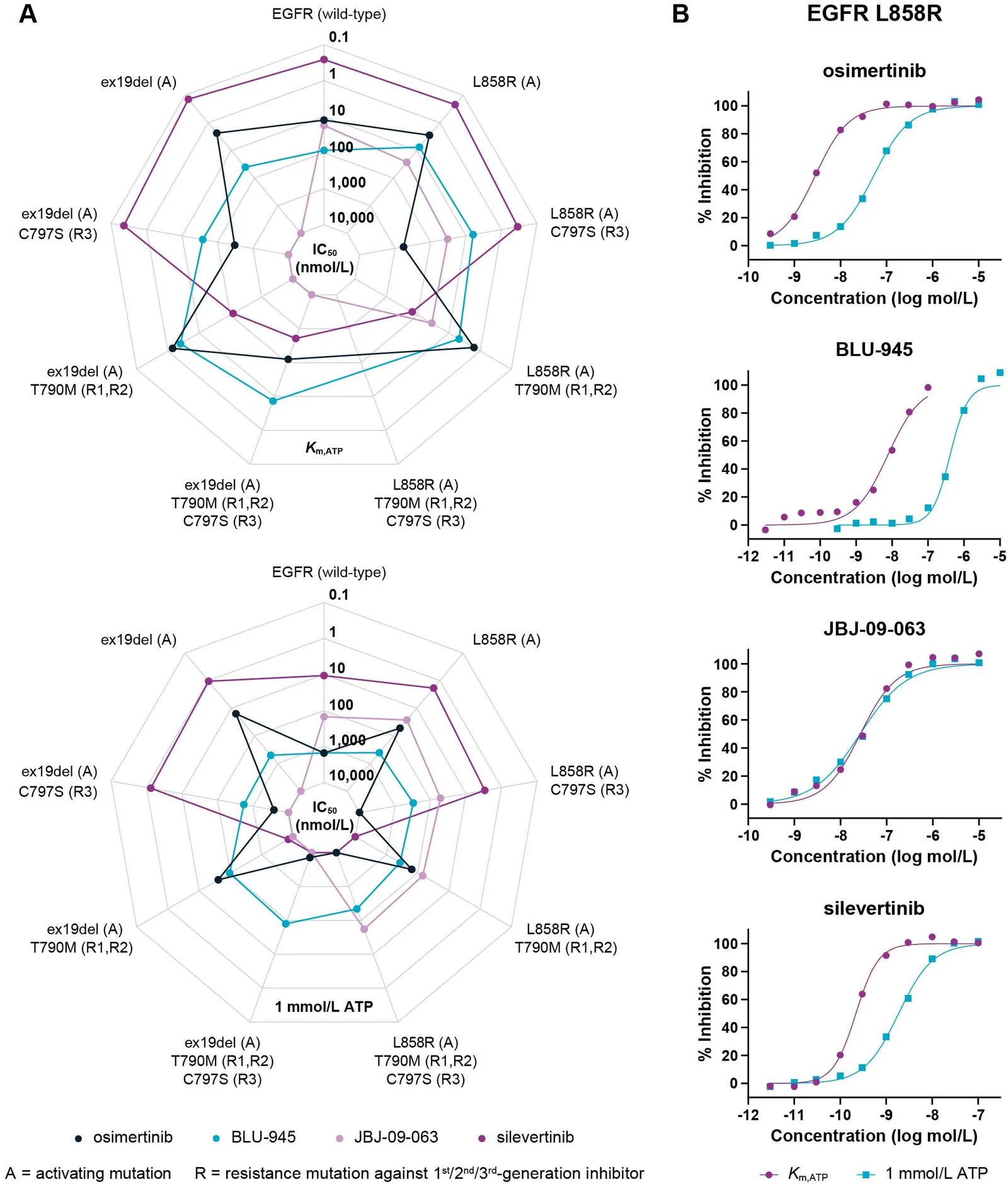
Biochemical profiling of EGFR inhibitors on wild-type and mutant EGFR. **(A)** Radar plots showing IC_50_ values determined at *K*_m,ATP_ and 1 mmol/L ATP. **(B)** Dose-response curves measured at *K*_m,ATP_ and 1 mmol/L ATP on EGFR L858R mutant.

**Table 2:** Inhibitory potencies (IC_50_, in nmol/L) of EGFR inhibitors in enzyme assays with wild-type and mutant forms of EGFR at *K*_m,ATP_ and 1 mmol/L ATP.

Condition	Inhibitor	Wild-type	L858R	ex19del	L858R + T790M	ex19del + T790M	L858R + C797S	ex19del + C797S	L858R + T790M + C797S	ex19del + T790M + C797S
	Effect of mutation^a^	–	A	A	A, R1, R2	A, R1, R2	A, R3	A, R3	A, R1-3	A, R1-3
*K*_m,ATP_	osimertinib	12.5^b^	2.82^b^	2.36^b^	1.57^b^	1.41^b^	574	304	n.d.^c^	125
	BLU-945	86.0	7.61	40.2	4.70	2.53	6.32	38.5	n.d.^c^	7.30
	JBJ-09-063	17.5	26.9	> 10,000	35.3	> 10,000	32.9	> 10,000	n.d.^c^	> 10,000
	silevertinib	0.265^b^	0.219^b^	0.144^b^	149^b^	122^b^	0.348	0.226	n.d.^c^	514
1 mmol/L ATP	osimertinib	1,510^b^	52.5^b^	15.8^b^	155^b^	41.0^b^	> 10,000	3,880	> 10,000	7,130
	BLU-945	1,490	409	504	367	95.2	306	550	217	79.2
	JBJ-09-063	148	26.6	> 10,000	69.0	> 10,000	52.2	> 10,000	54.9	> 10,000
	silevertinib	10.7^b^	1.86^b^	1.05^b^	> 10,000^b^	7190^b^	2.97	1.32	> 10,000	> 10,000

a Effect of EGFR mutation: A, activation; R1, first-generation EGFR inhibitor resistance; R2, second-generation EGFR inhibitor resistance; R3, osimertinib resistance.

b Inhibitor binds covalently to the EGFR variant and potency is therefore time-dependent.

c Not determined. This kinase was not available for assays conducted at *K*_m,ATP_ concentration, because the assay did not meet predefined assay criteria.

Osimertinib showed clear activity against EGFR forms carrying either the activating mutation L858R or ex19del, alone or in combination with the T790M mutation. Moderate to high selectivity for these mutants compared to wild-type EGFR was observed in the assays, which was most pronounced at 1 mmol/L ATP concentration. In line with its covalent targeting of the EGFR C797 residue, osimertinib inhibited EGFR variants carrying the C797S mutation with considerably reduced potency at both *K*_m,ATP_ and 1 mmol/L ATP.

BLU-945 had the most stable potency across the evaluated EGFR mutants at both ATP concentrations, with only some fluctuations observed for ex19del mutants. Among the fourth-generation EGFR inhibitors, BLU-945 showed the highest selectivity for most EGFR mutants relative to wild-type EGFR at *K*_m,ATP_ concentration, while the selectivity was reduced at 1 mmol/L ATP.

JBJ-09-063 was active on mutant proteins harboring the L858R mutation, but not the ex19del mutation, at both ATP conditions. It inhibited EGFR L858R and the corresponding double and triple mutants with similar potency at *K*_m,ATP_ and 1 mmol/L ATP concentrations (**Figure 2B**), suggesting a binding site distinct from the ATP-binding pocket. This is in line with previously reported data (16). JBJ-09-063 also showed activity on wild-type EGFR, although this was more pronounced at *K*_m,ATP_ compared to 1 mmol/L ATP concentration.

At both *K*_m,ATP_ and 1 mmol/L ATP, silevertinib demonstrated high potency against wild-type and mutant EGFR variants carrying the L858R or ex19del activating mutations, alone or in combination with the C797S osimertinib-resistance mutation. However, its potency was strongly reduced once the T790M mutation was present. Silevertinib was moderately selective against wild-type EGFR at 1 mmol/L ATP, while selectivity was completely lost at *K*_m,ATP_ concentration, although this may be related to potencies reaching the limit of detection. Silevertinib is described by its developers as an irreversible EGFR inhibitor (17, 18) and contains an acrylamide warhead designed to form a covalent bond with the cysteine at position 797. Notably, however, the potencies observed for activating EGFR mutants harboring the C797S mutation were comparable to those measured for variants containing only the activating mutations under both ATP conditions.

EGFR mutants harboring the osimertinib-resistance C797S mutation currently represent the most clinically relevant setting. Among the profiled inhibitors, silevertinib demonstrated the highest potency against the double mutants combining either activating EGFR mutation with the C797S mutation (**Figure 2A**. **Table 2**). This suggests that silevertinib could represent the most suitable therapeutic option of the studied fourth-generation inhibitors for patients who progress on osimertinib in the first-line setting. When the T790M mutation is present in addition to the C797S mutation, reflecting disease progression following second-line osimertinib treatment, the specific activating EGFR mutation is an important determinant of inhibitor potency. In the triple mutants, JBJ-09-063 was the most potent EGFR inhibitor in the presence of the L858R mutation, whereas BLU-945 was the most potent on the mutant harboring the exon 19 deletion (**Figure 2A**, **Table 2**).

### 3.2 Binding kinetics by surface plasmon resonance

The distinct profiles of the EGFR inhibitors were further explored using surface plasmon resonance (SPR). This technique allows direct measurement of inhibitor binding kinetics and affinity in the absence of ATP competition, thereby enabling characterization of the intrinsic inhibitor–protein interaction. Inhibitor binding was first studied with two EGFR mutant proteins: the L858R single mutant, representing a treatment naïve setting, and the L858R + C797S double mutant, representing disease progression after first-line osimertinib treatment. Since the inhibitors are known to have distinct binding modes (**Table 1**), two kinetic models were applied for curve fitting. For non-covalent interactions, a 1:1 binding model was used, whereas covalent interactions were fitted with a modified two-state reaction model that accounts for the two-step irreversible binding mechanism (see Methods).

The sensorgrams of osimertinib and silevertinib on EGFR L858R show strong binding with dissociation curves that quickly flatten, which is consistent with the irreversible, covalent binding mode of these inhibitors (**Figure 3A**). Determination of the *k*_inact_ and *K*_I_ parameters using the modified two-state reaction model revealed that the *k*_inact_/*K*_I_ ratio was highest for silevertinib. This indicates that silevertinib more efficiently inactivates EGFR L858R compared to osimertinib (**Table 3**), which is consistent with its higher potency measured in the biochemical assays (**Table 2**). BLU-945 and JBJ-09-063 additionally showed strong, reversible binding to this mutant with roughly similar binding affinities among the two inhibitors (**Figure 3A**, **Table 3**).

**Figure 3:**
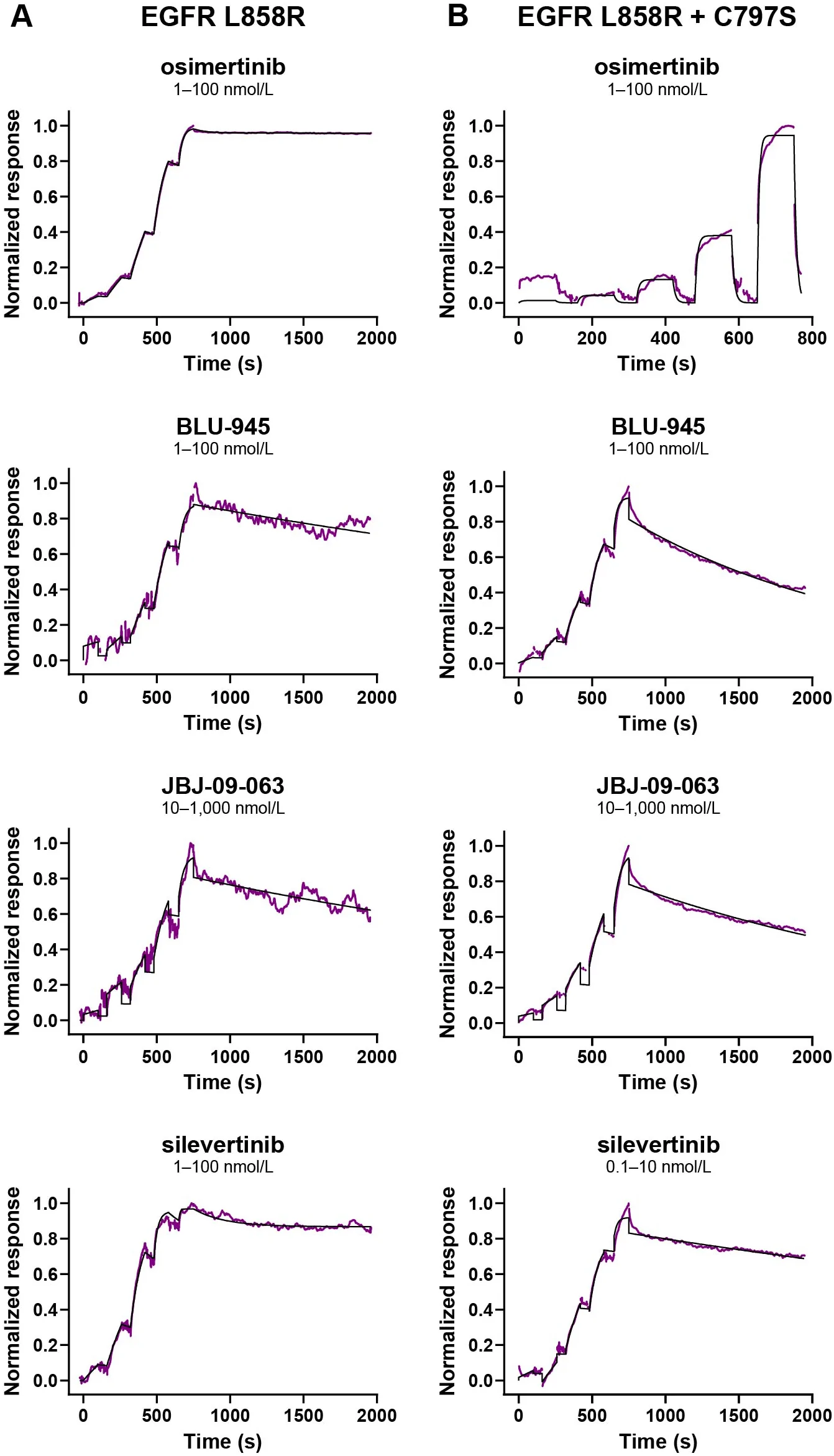
Binding kinetics of EGFR inhibitors on EGFR L858R and double mutant with C797S. Representative sensorgrams of single-cycle kinetics experiments with EGFR inhibitors and the cytoplasmic domain of EGFR **(A)** L858R and **(B)** L858R + C797S mutants are shown. Measured responses are shown in purple; fits obtained with a 1:1 binding model (for non-covalent interactions) or a modified two-state reaction model (for covalent interactions) are shown in black. The tested concentration range is shown for each inhibitor.

**Table 3:** Kinetic binding parameters of EGFR inhibitors on EGFR mutants.

EGFR mutant	Inhibitor	*k*_a_ (L mol^-1^ s^-1^)	*k*_d_ (s^-1^)	*K*_D_ (nmol/L)	*k*_inact_ (s^-1^)	*K*_I_ (nmol/L)	*k*_inact_/*K*_I_ (L µmol^-1^ s^-1^)	n
L858R	osimertinib^a^	5.69 × 10^5^	3.68 × 10^-3^		5.60 × 10^-3^	17.9	0.312	4
	BLU-945	1.18 × 10^5^	2.53 × 10^-4^	2.15				2
	JBJ-09-063	7.02 × 10^4^	2.17 × 10^-4^	3.09				1
	silevertinib^a^	4.73 × 10^6^	4.91 × 10^-3^		3.73 × 10^-3^	1.91	1.95	2
L858R + C797S	osimertinib	5.66 × 10^5^	1.16 × 10^-1^	205				1
	BLU-945	4.89 × 10^5^	7.18 × 10^-4^	1.47				3
	JBJ-09-063	2.82 × 10^4^	5.26 × 10^-4^	18.6				2
	silevertinib	5.50 × 10^6^	1.85 × 10^-4^	0.0337				3
L858R + T790M	osimertinib^a^	8.64 × 10^5^	4.73 × 10^-3^		4.38 × 10^-3^	13.5	0.324	7
	JBJ-09-063	7.55 × 10^3^	1.92 × 10^-4^	25.4				3
L858R + T790M + osimertinib (sat.)	JBJ-09-063	1.84 × 10^4^	3.06 × 10^-4^	34.6				2

*k*_a_ = association rate; *k*_d_ = dissociation rate; *K*_D_ = affinity; *k*_inact_ = inactivation rate; *K*_I_ = inactivation constant; n = number of independent experiments.

a Covalent inhibitors were fit with a modified two-state reaction model instead of the Langmuir 1:1 binding model (see Methods).

Presence of the C797S mutation greatly impaired binding of osimertinib, while strong binding of silevertinib was maintained despite loss of the cysteine residue required for its covalent binding (**Figure 3B**). Although the binding kinetics of silevertinib cannot be directly compared between the L858R and L858R + C797S variants due to the different inhibitor binding modes, the sub-nanomolar affinity of silevertinib to the double mutant is consistent with its sub-nanomolar biochemical potency at *K*_m,ATP_ concentration (**Tables 2**, **3**). The binding of BLU-945 and JBJ-09-063 was also retained in the presence of the C797S mutation, although with some loss of affinity for JBJ-09-063 (**Figure 3B**, **Table 3**).

The allosteric binding mode of JBJ-09-063 was additionally investigated in a competition experiment using SPR. As single agents, both osimertinib and JBJ-09-063 showed strong binding to the L858R + T790M EGFR double mutant (**Figure 4A** and **Table 3**). In the competition setup, a saturating concentration of osimertinib was added to the running buffer, followed by injection of a concentration range of osimertinib or JBJ-09-063 (**Figure 4B**). While no additional binding of osimertinib was observed, the binding of JBJ-09-063 to EGFR L858R + T790M was not affected by the saturated osimertinib binding (**Figure 4B**). This confirms that osimertinib and JBJ-09-063 bind to two distinct, non-overlapping sites of EGFR.

**Figure 4:**
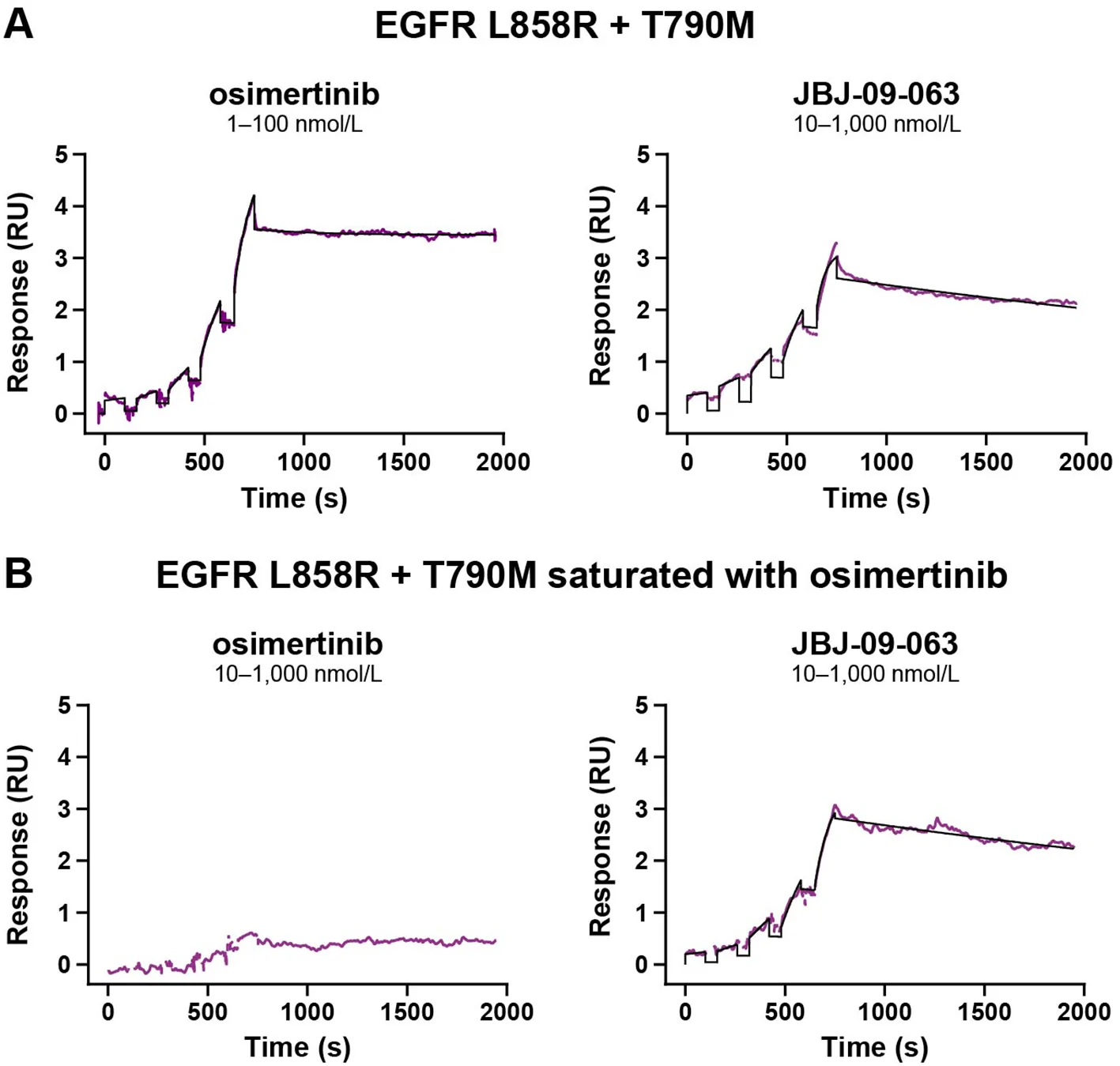
Competition binding experiment of JBJ-09-063 and osimertinib on EGFR L858R + T790M. Representative sensorgrams of a single-cycle kinetics experiment in the **(A)** absence and **(B)** presence of a saturating concentration of osimertinib (1 µmol/L) in the running buffer are shown. Measured responses are shown in purple; fits obtained with a 1:1 binding model (for non-covalent interactions) or a modified two-state reaction model (for covalent interactions) are shown in black. The tested concentration range is shown for each inhibitor.

### 3.3 Generation of an osimertinib-resistant C797S-mutant cell line

To assess the potency of the fourth-generation EGFR inhibitors in a relevant cellular model, the osimertinib-resistance mutation C797S was introduced into the NSCLC cell line NCI-H1975 using CRISPR/Cas9, as no publicly available cancer cell line harbors this mutation. The NCI-H1975 cell line carries both the EGFR L858R activating mutation and the EGFR T790M resistance mutation. Single-cell clones were derived from the transfected cell pool and, after confirming the presence of the EGFR C797S mutation, three cell clones were selected for further characterization. The clones were additionally characterized by assessing EGFR expression at the protein and RNA level. Immunoblotting showed that total EGFR levels were slightly higher in the C797S clones than in the parental cells, while phosphorylation levels were comparable (**Figure 5A**). Flow cytometry analysis revealed that the surface expression of EGFR was on average 2- to 2.5-fold higher in the clones than in the parental cells (**Figure 5B**). Notably, qPCR revealed no differences in EGFR mRNA levels between the clones and the parental cells (**Figure 5C**), indicating that the observed changes occurred at the post-transcriptional level.

**Figure 5:**
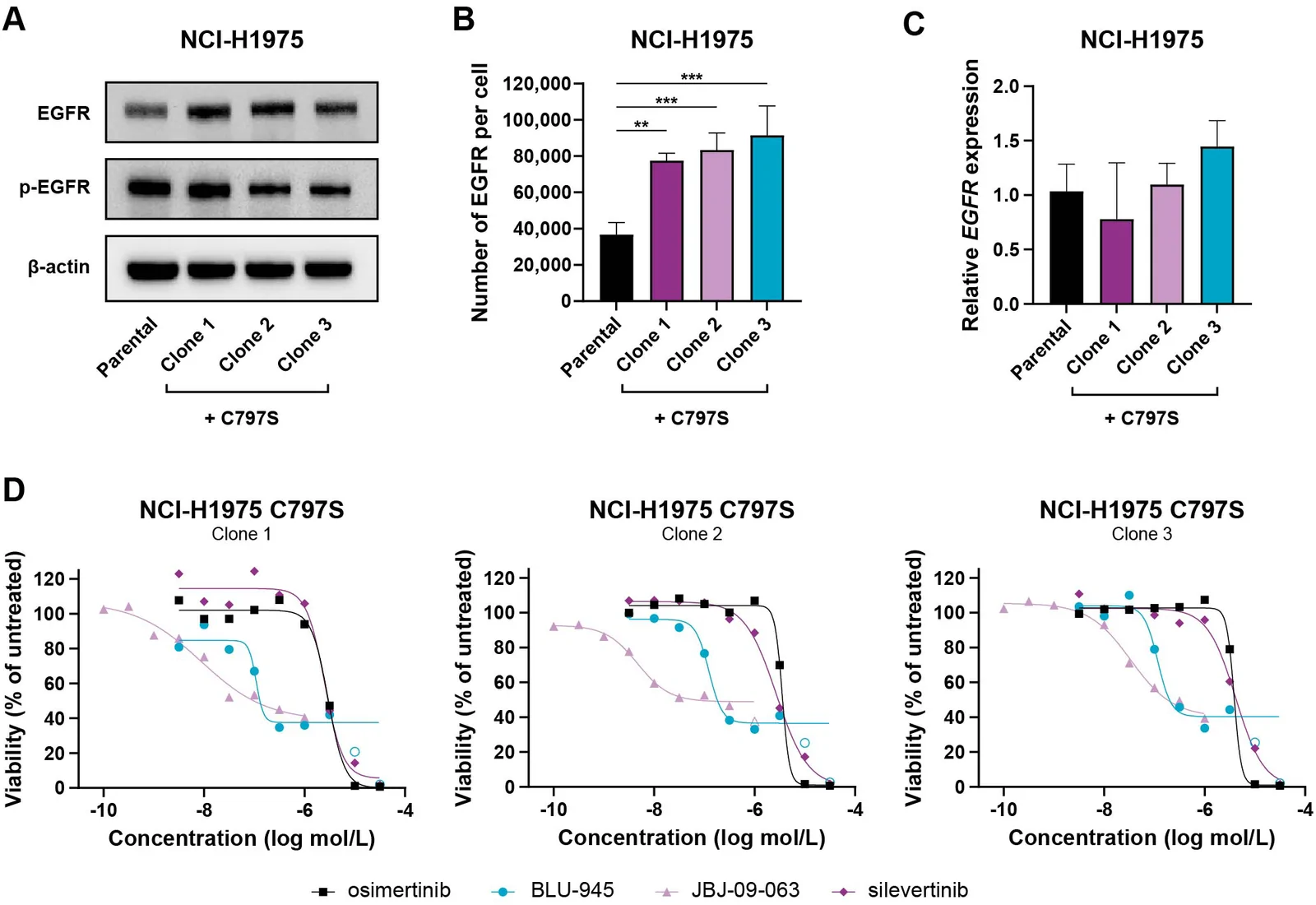
Characterization of a CRISPR-engineered NCI-H1975 cell line harboring the L858R + T790M + C797S triple mutation. Three osimertinib-resistant NCI-H1975 clones were generated via CRISPR/Cas9 gene editing and characterized with **(A)** immunoblotting of total and phosphorylated EGFR, **(B)** flow cytometry analysis of cell surface EGFR (*n* = 3), **(C)** qPCR of *EGFR* mRNA levels relative to *ACTB* mRNA levels and normalized to the parental (*n* = 3) and **(D)** cell viability assays (*n* = 1, average of technical duplicates is shown). Bars indicate mean + SD. Results of one-way ANOVA with Tukey’s multiple comparisons test are indicated in **(B)** **p < 0.01; ***p < 0.001. One-way ANOVA of gene expression results in **(C)** was not significant.

In viability assays, parental NCI-H1975 cells were inhibited by osimertinib with an IC_50_ of 11.7 nmol/L, while the IC_50_ values of osimertinib in the three cell clones were at least 250 times higher (**Table 4**, **Figure 5D**). Of the three fourth-generation inhibitors, both JBJ-09-063 and BLU-945 showed similar potencies across the double-mutant parental cell line and triple-mutant clones. JBJ-09-063 showed the strongest activity across these cell lines with a geometric mean IC_50_ value of 11.3 nmol/L, followed by BLU-945 with a geometric mean IC_50_ value of 118 nmol/L. In contrast, silevertinib showed limited activity in both the parental cell line and C797S-mutated clones. This finding is consistent with the reduced potency of silevertinib against T790M-mutant variants observed in the biochemical assays. Moreover, the potency of silevertinib was threefold reduced upon introduction of the C797S mutation.

**Table 4:** Inhibitory potencies (IC_50_, in nmol/L) of EGFR inhibitors in viability assays with *EGFR*-mutant lung cancer cell lines.

Cell line	EGFR mutation	osimertinib	BLU-945	JBJ-09-063	silevertinib
HCC827	Ex19del + amplification	5.09	347	15,700	0.355
HCC4006	Ex19del	17.7	754	13,400	0.514
NCI-H1650^a^	Ex19del	17.8	> 31,600	14,100	0.120
PC-9	Ex19del	13.9	1,850	6,500	0.839
11-18	L858R	18.8	722	109	2.73
NCI-H1975	L858R + T790M	11.7	126	12.0	755
NCI-H1975 C797S clone 1	L858R + T790M + C797S	2,950	109	8.64	2,530
NCI-H1975 C797S clone 2	L858R + T790M + C797S	3,550	123	4.68	2,710
NCI-H1975 C797S clone 3	L858R + T790M + C797S	3,860	116	33.8	4,130

a The NCI-H1650 cell line harbors a *PTEN* deletion as well.

### 3.4 Cancer cell panel profiling

The activity and selectivity of osimertinib and the fourth-generation EGFR inhibitors were further evaluated in a panel of lung cancer cell lines harboring *EGFR* mutations using cell viability assays (**Figure 6**). This panel included four cell lines with an activating ex19del mutation (HCC827, HCC4006, NCI-H1650 and PC-9), one with an L858R activating mutation (11–18), and the above-described cell line with both an L858R activating mutation and a T790M resistance mutation (NCI-H1975) (**Table 4**). In this lung cancer cell line panel, silevertinib showed the greatest potency in cell lines harboring only an activating EGFR mutation, followed by osimertinib (**Figure 6**, **Table 4**). Notably, osimertinib was the most effective compound in viability assays with NCI-H1975 cells, which is consistent with its known activity in overcoming T790M-mediated resistance (8). JBJ-09-063 showed activity only in the two cell lines harboring the L858R mutation, whereas BLU-945 demonstrated activity across cell lines carrying either the L858R mutation or exon 19 deletion.

**Figure 6:**
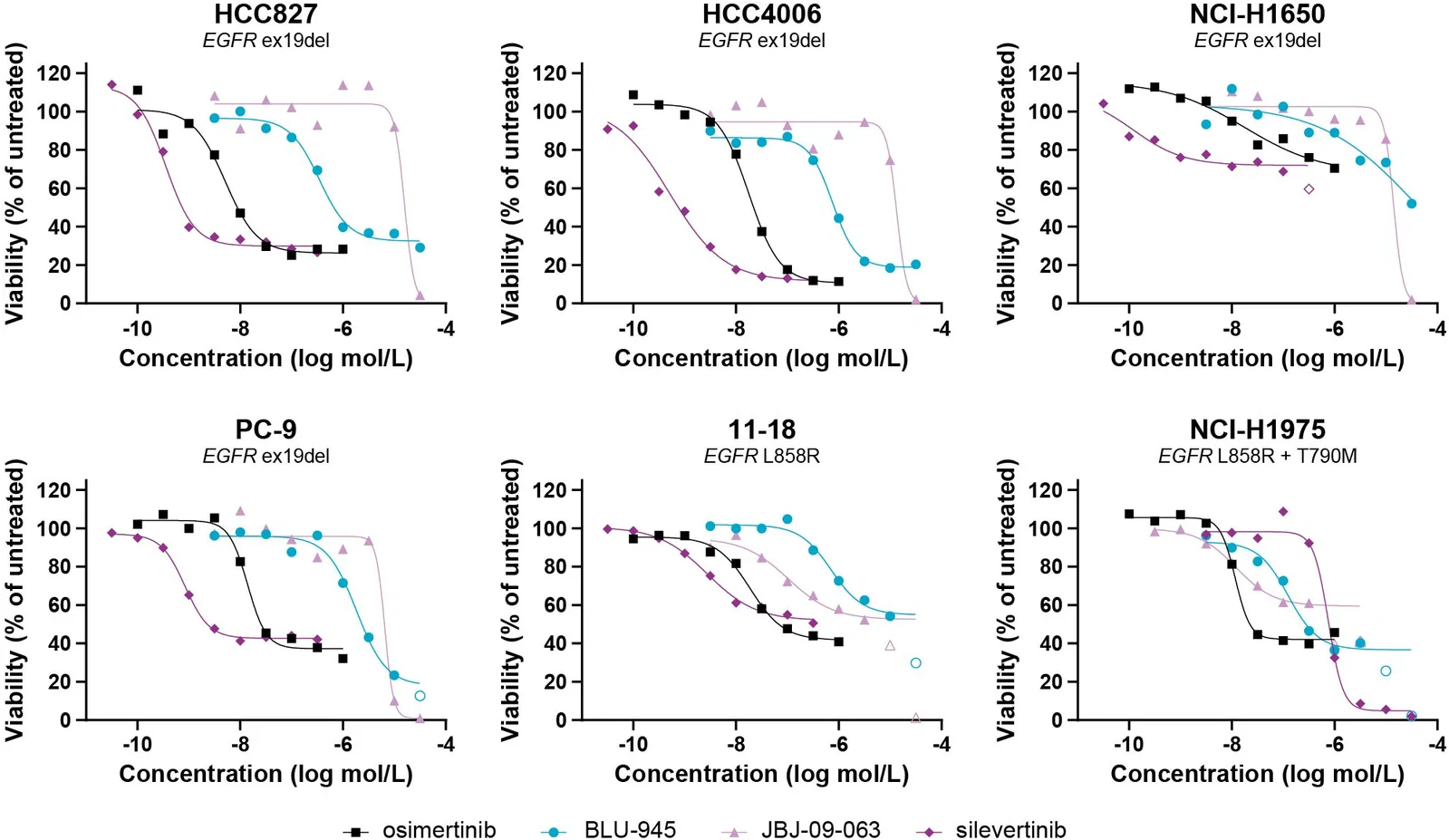
Dose-response curves of viability assays with EGFR inhibitors in six lung cancer cell lines harboring different *EGFR* mutations. Each datapoint is an average of technical duplicates.

Besides the EGFR ex19del, the NCI-H1650 cell line harbors a *PTEN* deletion (42). Inactivation of PTEN is associated with resistance against EGFR inhibitors (43). This is in line with the reduced efficacy, that is, the reduced maximal percentage effect of the EGFR inhibitors observed in this cell line compared to other cell lines harboring ex19del (**Figure 6**).

The four inhibitors were further profiled in 107 human cancer cell lines representing various tumor types (**Supplementary Table 4**) adding up to a total of 113 cell lines among which ten cell lines with *EGFR* mutations. In addition to the six cell lines described above harboring EGFR ex19del, L858R and T790M mutations, the panel included one cell line with a G719S mutation, one with an A289D mutation and two with an A289V mutation (**Supplementary Table 3**). G719S is an uncommon activating mutation localized in the kinase domain of EGFR. A289 is located in the extracellular domain and mutations at this residue are oncogenic (33, 35).

The relative targeting of *EGFR*-mutant *versus EGFR* wild-type cell lines was assessed by comparing the geometric mean IC_50_ values of the two groups. Osimertinib, BLU-945 and silevertinib demonstrated significantly greater inhibitory effects on *EGFR*-mutant cell lines compared to *EGFR* wild-type cell lines (*p* < 0.05). Among these inhibitors, silevertinib showed the strongest selectivity, as indicated by a 214-fold difference in IC_50_ values, followed by osimertinib (44-fold difference) and BLU-945 (9.3-fold difference), while JBJ-09-063 showed the smallest shift in IC_50_ (4.1-fold difference) (**Figure 7**). Since JBJ-09-063 is reported to require the L858R mutation for binding (15, 16) and the panel only consists of two EGFR L858R mutated cell lines, its selectivity may have been underestimated.

**Figure 7:**
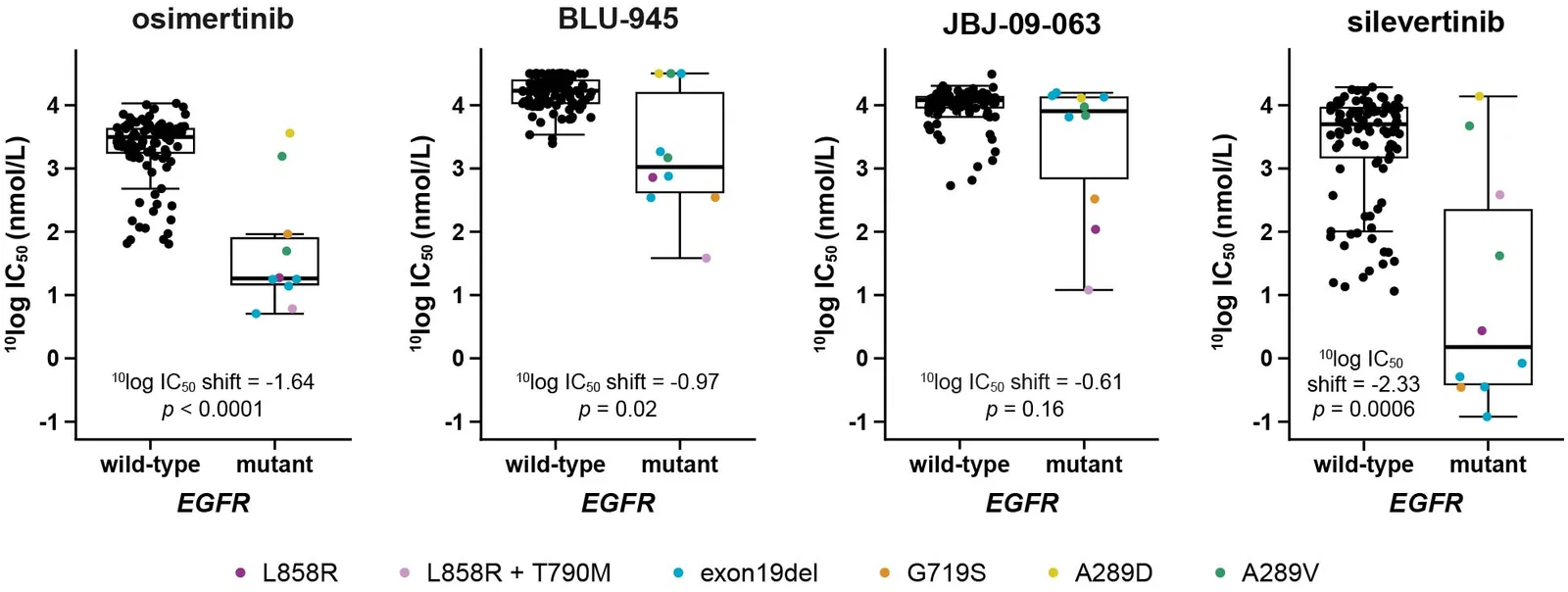
Cellular profiling of EGFR inhibitors in a panel of 113 human cancer cell lines. The box plots show the inhibitory potency of osimertinib and three fourth-generation EGFR inhibitors in viability assays with 103 cancer cell lines wild-type for *EGFR* and ten *EGFR*-mutant cell lines. The ten cell lines harboring mutations in *EGFR* are indicated with different colors. Wilcoxon rank-sum tests were used to determine the significance of the IC_50_ differences between *EGFR* wild-type and mutant cell lines.

Beyond evaluation of the inhibitor activities against common EGFR mutations, the large-scale cell line profiling allowed exploration of the potential of fourth-generation inhibitors to target uncommon EGFR mutations. Among the fourth-generation inhibitors, BLU-945 and silevertinib inhibited the proliferation of a cell line harboring the uncommon activating G719S mutation (SW48) with potencies comparable to those observed for the common activating EGFR mutations L858R and ex19del (**Figure 7**). In contrast, osimertinib showed reduced inhibitory activity in this cell line relative to cell lines harboring the common activating EGFR mutations. Osimertinib, BLU-945 and silevertinib additionally showed activity in one of the two cell lines harboring an A289V mutation. This indicates that among the fourth-generation EGFR inhibitors, BLU-945 and in particular silevertinib also exhibit activity against uncommon EGFR mutations.

## 4 Discussion

Selective targeting of *EGFR*-mutant NSCLC with small molecule kinase inhibitors has transformed the treatment paradigm of this deadly cancer. However, the emergence of acquired resistance limits the durability of responses, which has driven the development of successive generations of EGFR inhibitors.

The three fourth-generation inhibitors examined in this study were originally developed to overcome the EGFR C797S mutation, the most common on-target mechanism of resistance against third-generation inhibitor osimertinib in any line of therapy (13). When osimertinib was still used as second-line therapy, C797S most commonly emerged together with T790M. However, as osimertinib has since moved to the first-line setting in many countries (10 – 12), the double EGFR mutants L858R + C797S and ex19del + C797S are becoming clinically more relevant than the corresponding triple mutants L858R + T790M + C797S and ex19del + T790M + C797S.

Focusing specifically on these double EGFR mutants, all fourth-generation EGFR inhibitors showed binding to EGFR L858R + C797S in SPR experiments, with silevertinib demonstrating the strongest affinity followed first by BLU-945 and then JBJ-09-063. Similarly, in kinase assays, all fourth-generation inhibitors had an improved potency on the EGFR L858R + C797S mutant compared to osimertinib. In the context of the EGFR + ex19del mutant, both silevertinib and BLU-945 showed an increased potency compared to osimertinib, while JBJ-09-063 lost its potency, as expected based on literature (15, 16). Among the fourth-generation inhibitors, silevertinib showed the highest potency for both double EGFR mutants containing the C797S mutation with IC_50_ values in the sub-nanomolar to nanomolar range.

Contrarily to the other studied fourth-generation EGFR inhibitors, silevertinib is described as an irreversible EGFR inhibitor (17, 18) and is expected to form a covalent bond with the cysteine at position 797 through its acrylamide warhead (**Figure 1**). Nonetheless, silevertinib maintained sub-nanomolar affinity and potency on the C797S double mutants, highlighting its tight binding even in the absence of covalent bond formation with the cysteine at position 797. The mechanistic basis for this tight binding, whether related to high-affinity non-covalent binding or alternative covalent interactions, remains to be established. Structural studies could provide valuable insight for this. In contrast, silevertinib demonstrated a pronounced loss of activity in the presence of the T790M resistance mutation in both biochemical and cellular assays. In the biochemical assays, the potency of silevertinib substantially reduced when the T790M mutation was present together with an activating EGFR mutation, compared to the activating EGFR mutation alone. A similar effect was observed at a cellular level: the IC_50_ in NCI-H1975 cells (EGFR L858R + T790M) was more than 250-fold higher than that in 11–18 cells (EGFR L858R). These results are in line with previously reported data (23) and indicate that silevertinib is likely to be effective only in clinical situations in which the T790M mutation is not present.

JBJ-09-063 showed similar potencies at *K*_m,ATP_ and 1 mmol/L ATP in biochemical assays. Moreover, it was able to bind to mutant EGFR after saturation with osimertinib in an SPR competition experiment, indicating that it engages a binding site distinct from the ATP pocket. These results are consistent with published X-ray crystallographic data showing that JBJ-09-063 engages a binding site distinct from both the ATP-binding site and the osimertinib-binding pocket (15). It has been reported that JBJ-09-063 binds to a site that is formed as a result of the L858R mutation (15, 16). However, both our biochemical and cellular data reveal that JBJ-09-063 also exhibits measurable activity against wild-type EGFR. In the biochemical profiling at *K*_m,ATP_, JBJ-09-063 was the most potent on wild-type compared with the tested mutants, whereas at 1 mmol/L ATP a degree of selectivity toward mutant EGFR emerged. In addition, in the large cell line panel, the cell line harboring the uncommon EGFR activating mutation G719S and a small subset of the EGFR wild-type cell lines also displayed sensitivity to JBJ-09-063. Together, these findings suggest that, contrary to earlier statements (15, 16), the binding and activity of JBJ-09-063 are not restricted to L858R-containing EGFR variants but may also extend to wild-type EGFR under certain conditions.

Among the studied inhibitors, BLU-945 showed the broadest activity in the biochemical assays. Whereas the other inhibitors each displayed a marked loss of activity against some of the mutants, BLU-945 inhibited all evaluated mutants, with varying degrees of selectivity over wild-type EGFR. BLU-945 additionally inhibited the viability of cancer cell lines harboring some uncommon EGFR mutations, including G719S. While the clinical development of BLU-945 has been discontinued, this was done for strategic reasons and the compound has since been made available for out-licensing (press release 8 January 2024, www.blueprintmedicines.com, accessed 20 August 2026). Moreover, although final results of a completed phase 1/2 clinical trial have not been published, it is stated in the clinical trials database of the U.S. National Institute of Health (NIH) that the decision to terminate the trial was not related to safety concerns (NCT04862780, www.clinicaltrials.gov, accessed 20 August 2026). The potential application of BLU-945 in niche indications driven by uncommon EGFR mutants, such as G719S, should be investigated further by profiling of additional cell lines and testing on additional EGFR mutants in biochemical assays.

As none of the cell lines included in the large panel harbor the osimertinib-resistant C797S mutation, this alteration was introduced into the NCI-H1975 cell line using CRISPR/Cas9. In retrospect, this model is suboptimal for comparing fourth-generation EGFR inhibitors, as it intrinsically carries the T790M mutation and silevertinib has shown reduced biochemical activity against T790M-containing variants. Consistent with this, silevertinib already shows limited sensitivity in the parental cell line. Nevertheless, the relative potencies of the inhibitors observed in the CRISPR-engineered model are in line with the results from the biochemical assays with triple mutant EGFR L858R + T790M + C797S.

Overall, our study provides a comprehensive, side-by-side comparison of fourth-generation EGFR inhibitors. These findings highlight that the profiled fourth-generation EGFR inhibitors differ substantially in their activity landscapes, with implications for their optimal clinical deployment. It should be noted that the landscape of EGFR-targeting modalities extends beyond small-molecule kinase inhibitors and encompasses a range of biological approaches (44–47). Three monoclonal anti-EGFR antibodies and one bispecific antibody targeting EGFR and MET have been approved by the FDA for various cancer indications, including necitutumab (monospecific) and amivantamab (bispecific) for specific types of NSCLC (44, 45). While several anti-EGFR targeting antibody-drug conjugates (ADCs) are currently in clinical development (46), other chemical and biological approaches, including degraders and nanobodies, are being explored in the preclinical phase (47, 48). Progress in the development of these alternative modalities could directly influence the clinical positioning and therapeutic differentiation of fourth-generation EGFR inhibitors.

## Data Availability

The original contributions presented in the study are included in the article/**Supplementary Material**, further inquiries can be directed to the corresponding authors. One dataset presented in this study can be found in an online repository. The name of the repository and accession number can be found below: https://www.ebi.ac.uk/ena, PRJEB122979.
