# Supplementary material for: Comparative profiling of osimertinib and fourth-generation EGFR inhibitors on EGFR-mutant proteins and cell lines
Source: Front Oncol. 2026 Sep 21;16:1952701. doi: 10.3389/fonc.2026.1952701 (PMC13634975; doi:10.3389/fonc.2026.1952701)
Supplement: Supplementary file 1 [file DataSheet1.pdf]

Comparative profiling of osimertinib and fourth-generation EGFR inhibitors on EGFR-mutant proteins and cell lines

Janneke J.T.M. Melis[1]*, Kirsten J.W. Kevenaar[1], Jacob Ytsma[1], Daphne J.F. Kluitmans[1], Janneke M. van den End[1], Jeffrey J. Kooijman[1], Jonne L.K. van Eck[1], Esmee van den Bossche[1], Jeroen A.D.M. de Roos[1], Yusuke Kawase[2], Nicole Willemsen-Seegers[1], Yvonne Grobben[1] and Guido J.R. Zaman[1]*

[1]Oncolines B.V., Oss, The Netherlands

[2]Carna Biosciences, Inc., Kobe, Japan

*** Correspondence:**
Janneke J.T.M. Melis
janneke.melis@oncolines.com

Guido J.R. Zaman
info@oncolines.com

Supplementary Material

Supplementary Table S1

Source, concentration and apparent Michaelis constant of the proteins used in the kinase assays. The ATP concentration reflects the concentration of ATP used in the $K_{m,ATP}$ condition.

				$K_{m,ATP}$		1 mM ATP
**EGFR variant**	**Supplier**	**Cat. no.**	**Apparent Michaelis constant (µmol/L)**	**ATP concentration (µmol/L)**	**Enzyme concentration (nmol/L)**	**Enzyme concentration (nmol/L)**
Wild-type	Carna Biosciences	08-115	2.7	5	3.22	3.22
L858R	Carna Biosciences	08-502	9.8	10	1.87	0.93
Ex19del	Carna Biosciences	08-527	19	25	0.58	0.29
L858R + T790M	Carna Biosciences	08-510	1.9	2	4.38	2.19
Ex19del + T790M	Carna Biosciences	08-528	5.4	5	2.88	0.72
L858R + C797S	Carna Biosciences	08-563	4.1	5	2.48	1.03
Ex19del + C797S	Carna Biosciences	08-564	8.2	10	1.16	0.48
L858R + T790M + C797S	Carna Biosciences	08-559	0.85	-	-	2.62
Ex19del + T790M + C797S	Carna Biosciences	08-565	1.8	2	3.55	0.89

Supplementary Table S2 Overview of cancer cell lines used for viability assays.

Cell line	Tissue	Disease	Supplier	Cat. no.	RRID	Medium
11-18	Lung	Lung adenocarcinoma	RIKEN	RCB2093	CVCL_6659	RPMI 1640 + 10% BCS
5637	Urinary tract	Bladder carcinoma	ATCC	HTB-9	CVCL_0126	RPMI 1640 + 10% BCS
769-P	Kidney	Renal cell carcinoma	ATCC	CRL-1933	CVCL_1050	RPMI 1640 + 10% BCS
786-O	Kidney	Renal cell carcinoma	ATCC	CRL-1932	CVCL_1051	RPMI 1640 + 10% BCS
A-172	Central nervous system	Glioblastoma	ATCC	CRL-1620	CVCL_0131	DMEM + 10% BCS
A-204	Soft tissue	Embryonal rhabdomyosarcoma	ATCC	HTB-82	CVCL_1058	McCoy's + 10% BCS
A-375	Skin	Amelanotic melanoma	ATCC	CRL-1619	CVCL_0132	DMEM + 10% BCS
A-388	Skin	Skin squamous cell carcinoma	ATCC	CRL-7905	CVCL_1063	DMEM + 10% BCS
A-427	Lung	Lung adenocarcinoma	ATCC	HTB-53	CVCL_1055	EMEM + 10% BCS
A-431	Skin	Skin squamous cell carcinoma	ATCC	CRL-1555	CVCL_0037	DMEM + 10% FCS
A-498	Kidney	Renal cell carcinoma	ATCC	HTB-44	CVCL_1056	EMEM + 10% BCS
A-549	Lung	Lung adenocarcinoma	ATCC	CCL-185	CVCL_0023	F-12K + 10% BCS
A-704	Kidney	Renal cell carcinoma	ATCC	HTB-45	CVCL_1065	EMEM + 10% BCS
ACHN	Kidney	Papillary renal cell carcinoma	ATCC	CRL-1611	CVCL_1067	EMEM + 10% BCS
AN3-CA	Uterus	Endometrial adenocarcinoma	ATCC	HTB-111	CVCL_0028	EMEM + 10% BCS
AsPC-1	Pancreas	Pancreatic ductal adenocarcinoma	ATCC	CRL-1682	CVCL_0152	RPMI 1640 + 10% BCS
AU565	Breast	Breast adenocarcinoma	ATCC	CRL-2351	CVCL_1074	RPMI 1640 + 10% BCS
BT-20	Breast	Invasive breast carcinoma of no special type	ATCC	HTB-19	CVCL_0178	EMEM + 10% BCS
BT-474	Breast	Invasive breast carcinoma of no special type	ATCC	HTB-20	CVCL_0179	DMEM + 20% FCS + 10 µg/mL human insulin
BT-549	Breast	Invasive breast carcinoma of no special type	ATCC	HTB-122	CVCL_1092	RPMI 1640 + 10% BCS
BxPC-3	Pancreas	Pancreatic ductal adenocarcinoma	ATCC	CRL-1687	CVCL_0186	RPMI 1640 + 10% BCS
C-33 A	Cervix	Cervical squamous cell carcinoma	ATCC	HTB-31	CVCL_1094	EMEM + 10% BCS
CAL-27	Upper aerodigestive	Tongue squamous cell carcinoma	ATCC	CRL-2095	CVCL_1107	DMEM + 10% BCS
CCF-STTG1	Central nervous system	Astrocytoma	ATCC	CRL-1718	CVCL_1118	RPMI 1640 + 10% BCS
CCRF-CEM	Blood	Childhood T acute lymphoblastic leukemia	ATCC	CCL-119	CVCL_0207	RPMI 1640 + 10% BCS
COLO 205	Colorectal	Colon adenocarcinoma	ATCC	CCL-222	CVCL_0218	RPMI 1640 + 10% BCS
COLO 829	Skin	Cutaneous melanoma	ATCC	CRL-1974	CVCL_1137	RPMI 1640 + 10% BCS
Daoy	Central nervous system	Medulloblastoma	ATCC	HTB-186	CVCL_1167	EMEM + 10% BCS
DB	Lymphocyte	Diffuse large B-cell lymphoma germinal center B-cell type	ATCC	CRL-2289	CVCL_1168	RPMI 1640 + 10% BCS
DLD-1	Colorectal	Colon adenocarcinoma	ATCC	CCL-221	CVCL_0248	RPMI 1640 + 10% BCS
DoTc2 4510	Cervix	Cervical carcinoma	ATCC	CRL-7920	CVCL_1181	DMEM + 10% BCS

Supplementary Table S2 *(continued)*

Cell line	Tissue	Disease	Supplier	Cat. no.	RRID	Medium
DU145	Prostate	Prostate carcinoma	ATCC	HTB-81	CVCL_0105	EMEM + 10% BCS
DU4475	Breast	Breast carcinoma	ATCC	HTB-123	CVCL_1183	RPMI 1640 + 10% BCS
ES-2	Ovary	Ovarian clear cell adenocarcinoma	ATCC	CRL-1978	CVCL_3509	McCoy's + 10% BCS
FaDu	Upper aerodigestive	Hypopharyngeal squamous cell carcinoma	ATCC	HTB-43	CVCL_1218	EMEM + 10% BCS
G-361	Skin	Melanoma	ATCC	CRL-1424	CVCL_1220	McCoy's + 10% BCS
HCC4006	Lung	Lung adenocarcinoma	ATCC	CRL-2871	CVCL_1269	RPMI 1640 + 10% BCS
HCC827	Lung	Lung adenocarcinoma	ATCC	CRL-2868	CVCL_2063	RPMI 1640 + 10% BCS
HCT 116	Colorectal	Colon carcinoma	ATCC	CCL-247	CVCL_0291	McCoy's + 10% BCS
HCT 15	Colorectal	Colon adenocarcinoma	ATCC	CCL-225	CVCL_0292	RPMI 1640 + 10% BCS
HEC-6	Uterus	Endometrial endometrioid adenocarcinoma, variant with squamous differentiation	JCRB	JCRB1118	CVCL_2931	EMEM + 15% FCS
HL-60	Blood	Adult acute myeloid leukemia	ATCC	CCL-240	CVCL_0002	IMDM + 10% BCS
Hs 578T	Breast	Invasive breast carcinoma of no special type	ATCC	HTB-126	CVCL_0332	DMEM + 10% BCS
Hs 638	Central nervous system	Oligodendroglioma	ATCC	HTB-138	CVCL_0844	EMEM + 10% BCS
Hs 746.T	Gastric	Gastric adenocarcinoma	ATCC	HTB-135	CVCL_0333	DMEM + 10% BCS
Hs 766T	Pancreas	Pancreatic adenocarcinoma	ATCC	HTB-134	CVCL_0334	DMEM + 10% BCS
HT	Lymphocyte	Diffuse large B-cell lymphoma germinal center B-cell type	ATCC	CRL-2260	CVCL_1290	RPMI 1640 + 10% BCS
HT-1080	Soft tissue	Fibrosarcoma	ATCC	CCL-121	CVCL_0317	EMEM + 10% BCS
HuTu 80	Gastric	Duodenal adenocarcinoma	ATCC	HTB-40	CVCL_1301	EMEM + 10% BCS
J82	Urinary tract	Bladder carcinoma	ATCC	HTB-1	CVCL_0359	EMEM + 10% BCS
JAR	Uterus	Gestational choriocarcinoma	ATCC	HTB-144	CVCL_0360	RPMI 1640 + 10% BCS
Jurkat E6.1	Blood	Childhood T acute lymphoblastic leukemia	ATCC	TIB-152	CVCL_0367	RPMI 1640 + 10% BCS
K-562	Blood	Blast phase chronic myelogenous leukemia, BCR-ABL1 positive	ATCC	CCL-243	CVCL_0004	IMDM + 10% BCS
KATO III	Gastric	Gastric signet ring cell adenocarcinoma	ATCC	HTB-103	CVCL_0371	IMDM + 10% BCS
KG-1	Blood	Adult acute myeloid leukemia	ATCC	CCL-246	CVCL_0374	IMDM + 10% BCS
KLE	Uterus	Endometrial adenocarcinoma	ATCC	CRL-1622	CVCL_1329	DMEM/F12 + 10% BCS
Ku812	Blood	Chronic myelogenous leukemia, BCR-ABL1 positive	ATCC	CRL-2099	CVCL_0379	RPMI 1640 + 10% BCS

Supplementary Table S2 *(continued)*

Cell line	Tissue	Disease	Supplier	Cat. no.	RRID	Medium
LNCaP clone FGC	Prostate	Prostate carcinoma	ATCC	CRL-1740	CVCL_1379	RPMI 1640 + 10% BCS
LoVo	Colorectal	Colon adenocarcinoma	ATCC	CCL-229	CVCL_0399	F-12K + 10% BCS
LS174T	Colorectal	Colon adenocarcinoma	ATCC	CL-188	CVCL_1384	EMEM + 10% BCS
LS411N	Colorectal	Cecum adenocarcinoma	ATCC	CRL-2159	CVCL_1385	RPMI 1640 + 10% BCS
MCF-7	Breast	Invasive breast carcinoma of no special type	ATCC	HTB-22	CVCL_0031	EMEM + 10% BCS + 10 µg/mL human insulin
MeWo	Skin	Cutaneous melanoma	ATCC	HTB-65	CVCL_0445	EMEM + 10% BCS
MG-63	Bone	Osteosarcoma	ATCC	CRL-1427	CVCL_0426	EMEM + 10% BCS
MIA PaCa-2	Pancreas	Pancreatic ductal adenocarcinoma	ATCC	CRL-1420	CVCL_0428	DMEM + 10% BCS
MOLT-4	Blood	Adult T acute lymphoblastic leukemia	ATCC	CRL-1582	CVCL_0013	RPMI 1640 + 10% BCS
NCC-IT	Embryo	Testicular embryonal carcinoma	ATCC	CRL-2073	CVCL_1451	RPMI 1640 + 10% BCS
NCI-H1650	Lung	Minimally invasive lung adenocarcinoma	ATCC	CRL-5883	CVCL_1483	RPMI 1640 + 10% BCS
NCI-H1975	Lung	Lung adenocarcinoma	ATCC	CRL-5908	CVCL_1511	RPMI 1640 + 10% BCS
NCI-H460	Lung	Lung large cell carcinoma	ATCC	HTB-177	CVCL_0459	RPMI 1640 + 10% BCS
NCI-H661	Lung	Lung large cell carcinoma	ATCC	HTB-183	CVCL_1577	RPMI 1640 + 10% BCS
NCI-H82	Lung	Lung small cell carcinoma	ATCC	HTB-175	CVCL_1591	RPMI 1640 + 10% BCS
OVCAR-3	Ovary	High grade ovarian serous adenocarcinoma	ATCC	HTB-161	CVCL_0465	RPMI 1640 + 10% BCS
PA-1	Ovary	Ovarian mixed germ cell tumor	ATCC	CRL-1572	CVCL_0479	EMEM + 10% BCS
PC-3	Prostate	Prostate carcinoma	ATCC	CRL-1435	CVCL_0035	F-12K + 10% BCS
PC-9	Lung	Lung adenocarcinoma	RIKEN	RCB4455	CVCL_B260	RPMI 1640 + 10% BCS
PFSK-1	Central nervous system	Primitive neuroectodermal tumor	ATCC	CRL-2060	CVCL_1642	RPMI 1640 + 10% BCS
RD	Soft tissue	Embryonal rhabdomyosarcoma	ATCC	CCL-136	CVCL_1649	DMEM + 10% BCS
Rh30	Soft tissue	Alveolar rhabdomyosarcoma	ATCC	CRL-2061	CVCL_0041	RPMI 1640 + 10% BCS
RKO	Colorectal	Colon carcinoma	ATCC	CRL-2577	CVCL_0504	EMEM + 10% BCS
RL	Lymphocyte	Diffuse large B-cell lymphoma	ATCC	CRL-2261	CVCL_1660	RPMI 1640 + 10% BCS
RL95-2	Uterus	Endometrial adenosquamous carcinoma	ATCC	CRL-1671	CVCL_0505	DMEM/F12 + 10% BCS
RPMI-7951	Skin	Melanoma	ATCC	HTB-66	CVCL_1666	EMEM + 10% BCS
RS4;11	Blood	Adult B acute lymphoblastic leukemia	ATCC	CRL-1873	CVCL_0093	RPMI 1640 + 10% BCS
RT-4	Urinary tract	Bladder carcinoma	ATCC	HTB-2	CVCL_0036	McCoy's + 10% BCS
SHP-77	Lung	Lung small cell carcinoma	ATCC	CRL-2195	CVCL_1693	RPMI 1640 + 10% BCS

Supplementary Table S2 *(continued)*

Cell line	Tissue	Disease	Supplier	Cat. no.	RRID	Medium
SK-BR-3	Breast	Breast adenocarcinoma	ATCC	HTB-30	CVCL_0033	McCoy's + 10% BCS
SK-N-AS	Peripheral nervous system	Neuroblastoma	ATCC	CRL-2137	CVCL_1700	DMEM + 10% BCS
SK-N-FI	Peripheral nervous system	Neuroblastoma	ATCC	CRL-2142	CVCL_1702	DMEM + 10% BCS
SNU-5	Gastric	Gastric adenocarcinoma	ATCC	CRL-5973	CVCL_0078	IMDM + 10% BCS
SNU-C2B	Colorectal	Cecum adenocarcinoma	ATCC	CCL-250	CVCL_1710	RPMI 1640 + 10% BCS
SR	Lymphocyte	Anaplastic large cell lymphoma, ALK-positive	ATCC	CRL-2262	CVCL_1711	RPMI 1640 + 10% BCS
SU-DHL-1	Lymphocyte	Anaplastic large cell lymphoma, ALK-positive	ATCC	CRL-2955	CVCL_0538	RPMI 1640 + 10% BCS
SU-DHL-6	Lymphocyte	Diffuse large B-cell lymphoma germinal center B-cell type	ATCC	CRL-2959	CVCL_2206	RPMI 1640 + 10% BCS
SUP-T1	Blood	Childhood T lymphoblastic lymphoma	DSMZ	ACC140	CVCL_1714	RPMI 1640 + 10% BCS
SW48	Colorectal	Colon adenocarcinoma	ATCC	CCL-231	CVCL_1724	RPMI 1640 + 10% BCS
SW480	Colorectal	Colon adenocarcinoma	ATCC	CCL-228	CVCL_0546	RPMI 1640 + 10% BCS
SW620	Colorectal	Colon adenocarcinoma	ATCC	CCL-227	CVCL_0547	RPMI 1640 + 10% BCS
SW626	Colorectal	Colon adenocarcinoma	ATCC	HTB-78	CVCL_1725	RPMI 1640 + 10% BCS
SW837	Colorectal	Rectal adenocarcinoma	ATCC	CCL-235	CVCL_1729	RPMI 1640 + 10% BCS
SW872	Soft tissue	Liposarcoma	ATCC	HTB-92	CVCL_1730	RPMI 1640 + 10% BCS
SW900	Lung	Lung squamous cell carcinoma	ATCC	HTB-59	CVCL_1731	RPMI 1640 + 10% BCS
SW948	Colorectal	Colon adenocarcinoma	ATCC	CCL-237	CVCL_0632	RPMI 1640 + 10% BCS
SW982	Soft tissue	Biphasic synovial sarcoma	ATCC	HTB-93	CVCL_1734	RPMI 1640 + 10% BCS
T24	Urinary tract	Bladder carcinoma	ATCC	HTB-4	CVCL_0554	McCoy's + 10% BCS
T98G	Central nervous system	Glioblastoma	ATCC	CRL-1690	CVCL_0556	EMEM + 10% BCS
TCCSUP	Urinary tract	Bladder carcinoma	ATCC	HTB-5	CVCL_1738	EMEM + 10% BCS
THP-1	Blood	Childhood acute monocytic leukemia	ATCC	TIB-202	CVCL_0006	RPMI 1640 + 10% BCS
TT	Thyroid	Hereditary thyroid gland medullary carcinoma	ATCC	CRL-1803	CVCL_1774	F-12K + 10% BCS
U-118MG	Central nervous system	Astrocytoma	ATCC	HTB-15	CVCL_0633	DMEM + 10% BCS
U2OS	Bone	Osteosarcoma	ATCC	HTB-96	CVCL_0042	McCoy's + 10% BCS
U-87MG ATCC	Central nervous system	Glioblastoma	ATCC	HTB-14	CVCL_0022	EMEM + 10% BCS
VA-ES-BJ	Soft tissue	Epithelioid sarcoma	ATCC	CRL-2138	CVCL_1785	DMEM + 10% BCS

BCS, bovine calf serum; FCS, fetal calf serum

Supplementary Table S3

Overview of CRISPR/Cas9 gene editing details in NCI-H1975 cells.

**Gene Name**	EGFR
**Ensembl transcript ID**	ENST00000275493.7
**Guide RNA sequence**	AUAGUCCAGGAGGCAGCCGA
**Guide RNA cut location**	Chr 7: 55,181,396
**Donor sequence (5’-3’)**	GTACTGGGAGCCAATATTGTCTTTGTGTTCCCGGACATAGTCCAG GAGGCTGCCGAAGGGCATGAGCTGCGTGATGAGCTGCACGGTGGAGGTGAGGCAGA
**PCR primers**	FOR Primer (5’-3’): TTTGCAGGCACAGCTTTTCC<br>REV Primer (5’-3’): CTCTTGCTATCCCAGGAGCG
**Sequencing primer**	Reverse

Supplementary Table S4

Frequency of *EGFR* gene mutations in the parental NCI-H1975 cell line and three osimertinib-resistant clones generated by introduction of the C797S mutation via CRISPR/Cas9 gene editing.

	**L858R**	**T790M**	**C797S**
Parental	65%	68%	0%
Clone 1	62%	45%	100%
Clone 2	63%	43%	100%
Clone 3	66%	48%	100%

Supplementary Table S5

Overview of *EGFR*-mutant cell lines used in this study.

Cell line	Mutation	Variant allele frequency (VAF)	Reference mutation status and VAF
HCC4006	Ex19del	0.74	DepMap (33)
NCI-H1650	Ex19del	0.63	DepMap (33)
PC-9	Ex19del	0.84	DepMap (33)
HCC827	Ex19del + amplification	0.96	DepMap (33)
11-18	L858R	0.57	Conlon *et al.* (28)
NCI-H1975	L858R + T790M	0.63	Conlon *et al.* (28)
Hs 683	A289D	0.38	DepMap (33)
HEC-6	A289V	0.38	Conlon *et al.* (28)
RL95-2	A289V	0.12	Conlon *et al.* (28)
SW48	G719S	0.33	Conlon *et al.* (28)

Supplementary Figures

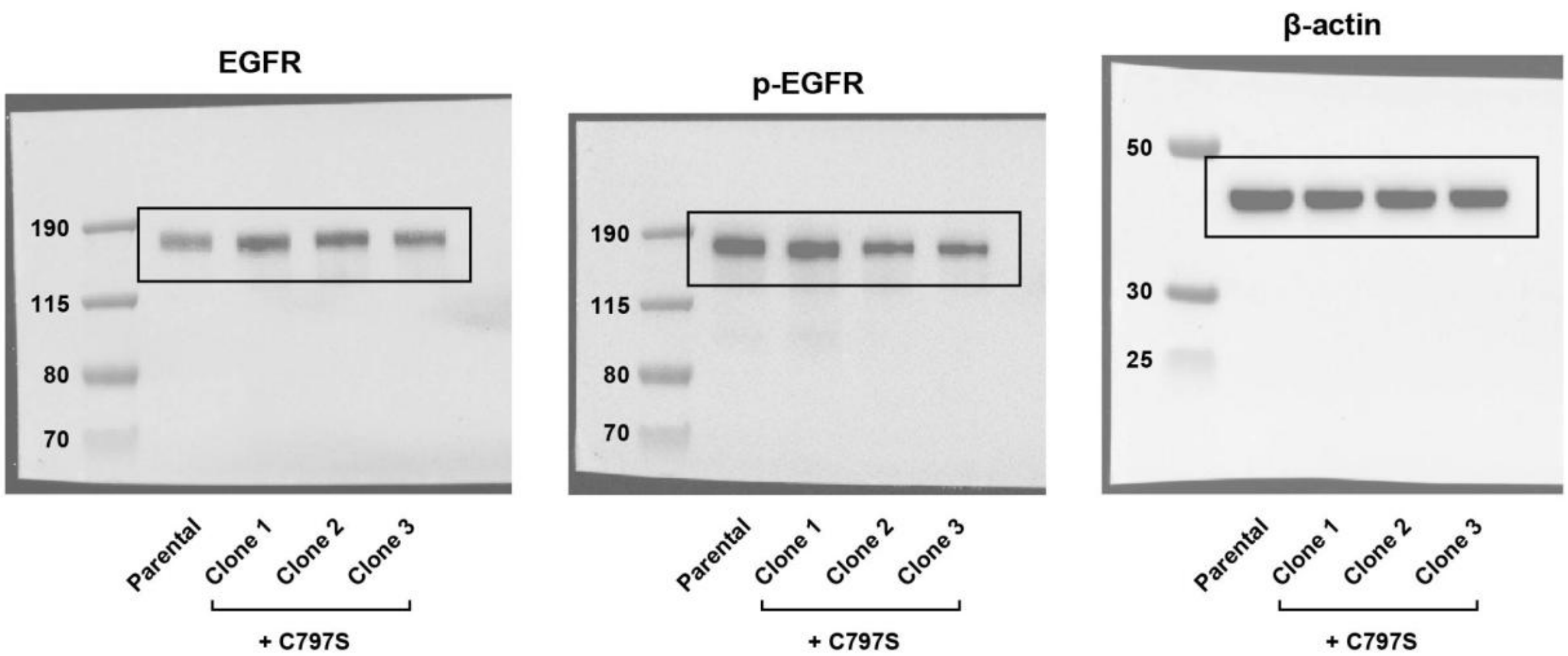

Supplementary Figure S1

Original images of the Western blot analysis. Overlays of chemiluminescent and colorimetric photos were made.
